# Feasibility of transference of inoculation-related technologies: A case study of evaluation of soybean rhizobial strains under the agro-climatic conditions of Brazil and Mozambique

**DOI:** 10.1016/j.agee.2017.06.037

**Published:** 2018-07-01

**Authors:** Amaral Machaculeha Chibeba, Stephen Kyei-Boahen, Maria de Fátima Guimarães, Marco Antonio Nogueira, Mariangela Hungria

**Affiliations:** a Universidade Estadual de Londrina (UEL), C.P. 10.011, 86.057-970, Londrina, PR, Brazil; b Embrapa Soja, C.P. 231, 86001-970, Londrina, PR, Brazil; c International Institute of Tropical Agriculture (IITA), P.O. Box 709, Nampula, Mozambique

**Keywords:** Biological nitrogen fixation, *Glycine max*, *Bradyrhizobium*, Inoculation, N-fertilizer, Sustainability

## Abstract

The soybean-*Bradyrhizobium* symbiosis can be very effective in fixing nitrogen and supply nearly all plant's demand on this nutrient, obviating the need for N-fertilizers. Brazil has been investing in research and use of inoculants for soybean for decades and with the expansion of the crop in African countries, the feasibility of transference of biological nitrogen fixation (BNF) technologies between the continents should be investigated. We evaluated the performance of five strains (four Brazilian and one North American) in the 2013/2014 and 2014/2015 crop seasons in Brazil (four sites) and Mozambique (five sites). The experimental areas were located in relatively similar agro-climatic regions and had soybean nodulating rhizobial population ranging from ≪ 10 to 2 × 10^5^ cells g^−1^ soil. The treatments were: (1) NI, non-inoculated control with no N-fertilizer; (2) NI + N, non-inoculated control with 200 kg of N ha^−1^; and inoculated with (3) *Bradyrhizobium japonicum* SEMIA 5079; (4) *B. diazoefficiens* SEMIA 5080; (5) *B. elkanii* SEMIA 587; (6) *B. elkanii* SEMIA 5019; (7) *B. diazoefficiens* USDA 110; (8) SEMIA 5079 + 5080 (only tested in Brazil). The best inoculation treatments across locations and crop seasons in Brazil were SEMIA 5079 + 5080, SEMIA 5079 and USDA 110, with average grain yield gains of 4–5% in relation to the non-inoculated treatment. SEMIA 5079, SEMIA 5080, SEMIA 5019 and USDA 110 were the best strains in Mozambique, with average 20–29% grain yield gains over the non-inoculated treatment. Moreover, the four best performing strains in Mozambique resulted in similar or better yields than the non-inoculated + N treatment, confirming the BNF as an alternative to N-fertilizers. The results also confirm the feasibility to transfer soybean inoculation technologies between countries, speeding up the establishment of sustainable cropping systems.

## Highlights

• Soybean N demand can be fulfilled by biological nitrogen fixation (BNF).
• *Bradyrhizobium* strains from Brazil and USA were tested in Brazil and Mozambique.
• Inoculation resulted in grain yield gains of 4–5% in Brazil and 20–29% in Mozambique.
• Transference of BNF technologies is feasible, speeding up the production system.
• Exotic soybean *Bradyrhizobium* strains can highly benefit soybean in Mozambique.

## 1 Introduction

Soybean [*Glycine max* (L.) Merrill] has potential to play a major role in responding to global food insecurity that results from mounting demographic pressures. The world population is projected to grow beyond 10 billion by 2100 (Gerland et al., 2014), and much of the increase will occur in Africa (Cleland, 2013), where hunger is already a threat. With high concentration of seed protein (40%), that provides all essential amino acids in sufficient amounts for human health, and high seed oil content (20%), soybean has many uses, encompassing human food, animal feed and biofuels. Moreover, soybean offers a number of advantages in sustainable cropping systems, including the ability to symbiotically fix atmospheric nitrogen (N_2_), which obviates the reliance on N-fertilizers.

Numerous reports testify that when soybean is grown for the first time in new areas outside Southeast Asia, its centre of origin and domestication, it generally requires inoculation with exotic strains (Pulver et al., 1985, Hungria et al., 2006b, Abaidoo et al., 2007, Giller et al., 2011, Hungria and Mendes, 2015). In Africa, where the distribution of inoculants represents another limitation, a strategy consisting in the use of promiscuous soybean genotypes—capable of forming nodules with indigenous rhizobia (Pulver et al., 1985, Abaidoo et al., 2007, Tefera, 2011)—has been used for decades; this strategy would be useful especially for smallholder farmers with no access to inoculants (Mpepereki et al., 2000). Nevertheless, with cropping intensification, the search for soybean genotypes with higher yield potential but requiring inoculation is scaling up.

Soybean response to inoculation is dependent on a number of environmental factors including soil N availability (Thies et al., 1991, Singleton et al., 1992), temperature (Hungria and Vargas, 2000), pH (Giller, 2001, Al-Falih, 2002), salinity (Zahran, 2010), P availability (Ronner et al., 2016) and, more importantly, indigenous rhizobial populations (Thies et al., 1992, Osunde et al., 2003). Very often, elite inoculant strains fail to overcome the competition barrier for nodule occupancy imposed by indigenous or naturalized rhizobia (Thies et al., 1992, Streeter, 1994, Vlassak et al., 1997, Al-Falih, 2002), most times ineffective but very competitive and already adapted to the environment (Streeter, 1994; Al-Falih, 2002; Grönemeyer et al., 2014). However, strong evidence of inoculation success in areas with high rhizobial population, of 10^3^–10^6^ cells g^−1^ of soil, has been published from Brazil (Hungria et al., 2005, Hungria et al., 2006a, Hungria et al., 2013, Campo et al., 2009, Hungria and Mendes, 2015), opening a window for inoculation research in other geographic regions.

Ecological studies on rhizobia have established that exogenous inoculant strains undergo genetic changes (Schloter et al., 2000, Barcellos et al., 2007) and may acquire superior competitive abilities as they become naturalized (Dunigan et al., 1984, Dowdle and Bohlool, 1987, Hungria and Mendes, 2015). The success of inoculation and nitrogen fixation on soybean in Brazil is chiefly ascribed to strain selection programs that took place for over half a century, in addition to the development of proper inoculation methods (Hungria et al., 2006a, Hungria and Mendes, 2015). On the contrary, in Mozambique soybean is a relatively new crop practiced primarily with promiscuous varieties without inoculation (Gyogluu et al., 2016). In recent years, nevertheless, the increased demand for soybean grain to supply the chicken industry and for export (Dias and Amane, 2011) has led to search for more productive non-promiscuous genotypes, which are generally responsive to commercial inoculants. The agro-climatic conditions of the soybean production areas in Mozambique are similar to the major soybean growing areas in Brazil, raising the question on whether the inoculant strains that perform well on a variety of agro-climatic zones in Brazil could be successfully transferred to Mozambique, saving time, labour and money.

The objective of this study was to compare the performance of four elite *Bradyrhizobium* strains from Brazil (SEMIA 587, 5019, 5079, and 5080) and another strain adopted as standard inoculant in many African countries (USDA 110) in trials carried out with non-promiscuous soybean genotypes in Brazil (four sites) and Mozambique (five sites).

## 2 Material and methods

### 2.1 Sites description: location, climate and soil characterization

Climate and soil classification (Table 1), soil chemical properties and rhizobial counts (Table 2), rainfall (Supplementary Table 1) and temperature (Supplementary Table 2) data are presented on the indicated tables. Sixty days prior to commencing the experiments, 20 soil sub-samples (0–20 cm) were collected at each site to evaluate biological, physical and chemical properties. Rhizobial population sizes were estimated by the most probable number (MPN) method (Vincent, 1970) with soybean cultivar BMX Potência RR (in Brazil) or Storm (in Mozambique). Silt, sand and clay fractions were determined by the hydrometer method (Kilmer and Alexander, 1949). In Mozambique, soil pH was determined in H_2_O (1/2; soil/water) 60 min after agitation. Ca, Mg, Al, K and P were determined by inductively coupled plasma optical emission spectroscopy (ICP-OES) after extraction with Mehlich-3 (Sims, 1989). In Brazil, chemical analyses were performed as described by Sparks et al. (1996). Soil pH was determined in 0.01 mol L^−1^ CaCl_2_ (1/2.5; soil/solution). Exchangeable Al, Mg and Ca were extracted with 1 mol L^−1^ KCl (1:10; soil/solution) after agitation for 10 min, P and K were extracted with Mehlich-1 after 10 min agitation. Aluminum was determined by titration with 0.015 mol L^−1^ standardized NaOH with indicator bromothymol blue, K was determined in a flame photometer, Ca and Mg were determined in an atomic absorption spectrophotometer, and P by the molybdenum-blue method with C_6_H_8_O_6_ as reducing agent. In both countries soil organic carbon (SC) was determined by the Walkley-Black chromic acid wet oxidation method (Walkley and Black, 1934) and soil organic matter (SOM) was obtained considering SOM = 1.724 × SC.

**Table 1:** Location, climate, soil type and textural class of the experimental sites.

Experimental site	Georeference			Climate^1^	Soil type^2^	Textural class^3^
	Latitude	Longitude	Altitude (m)			
Brazil						
Londrina	23°11′S	51°11′W	620	*Cfa*	Rhodic Ferralsols	Clay
Maracaí	22°36′S	50°40′W	475	*Cfa*	Ferric Luvisols	Sandy
Ponta Grossa	25°13′S	50°01′W	880	*Cfb*	Orthic Ferralsols	Sandy clay loamy
Rio Verde	17°47′S	50°54′W	730	*Aw*	Acric Ferralsols	Sandy clay
Mozambique						
Muriaze	15°16′S	39°19′E	363	*Aw*	Ferric Luvisols	Sandy clay loamy
Nkhame	14°38′S	33°59′E	1115	*Cwa*	Orthic Ferralsols	Sandy loamy
Ntengo	14°33′S	34°11′E	1225	*Cwa*	Orthic Ferralsols	Clay
Ruace	15°08′S	36°25′E	673	*Cwa*	Rhodic Ferralsols	Sandy
Sussundenga	19°19′S	33°15′E	611	Cwa	Rhodic Ferralsols	Sandy

1 Based on Köppen-Geiger climate classification (Pidwirny, 2011).

2 Based on FAO soil classification (FAO, 2016).

3 Based on USDA textural soil classification (USDA, 1987).

**Table 2:** Rhizobial count (MPN g^−1^ soil), soil chemical properties (pH, CaCl_2_; soil organic matter, g dm^−3^; Organic P, mg dm^−3^; Exchangeable K, Ca and Mg, cmol_c_ dm^−3^; exchangeable acidity, cmol_c_ dm^−3^) and soil granulometry (silt, sand and clay, g kg^−1^) of the locations where the field trials were conducted in the 2013/2014 and 2014/2015 crop seasons in Brazil and Mozambique.

Soil characteristic	Experimental sites in Brazil^1^					Experimental sites in Mozambique^2^									
	2013/14 season			2014/15 season		2013/14 crop season					2014/15 crop season				
	Lon	Mar	Rio	Lon	Pon	Mur	Nkh	Nte	Rua	Sus	Mur	Nkh	Nte	Rua	Sus
Rhizobia (MPN g^−1^ soil)	2 × 10^5^	≪10	≪10	5 × 10^5^	3 × 10^4^	≪10	1 × 10^3^	75	1 × 10^3^	≪10	na^9^	na	na	na	na
pH^3^ (CaCl_2_)	5.6	5.4	5.0	5.7	5.5	5.9	5.5	6.3	4.9	5.4	5.9	5.5	5.3	5.3	5.5
SOM^4^ (g dm^−3^)	23.56	8.41	50.74	23.79	30.86	41.38	12.41	22.80	13.53	11.38	27.41	25.17	21.90	18.10	16.21
Organic P (mg dm^−3^)	22.01	6.57	2.45	41.00	2.55	13.20	27.60	7.96	22.40	4.12	3.94	19.10	2.17	28.50	16.50
K (cmol_c_ dm^−3^)	0.61	0.05	0.17	1.13	1.11	0.65	0.22	2.02	0.27	0.22	0.56	0.31	0.56	0.38	0.16
Ca (cmol_c_ dm^−3^)	4.47	1.20	3.46	5.05	3.02	9.45	3.11	8.70	2.12	3.38	7.25	3.67	6.20	3.61	2.45
Mg (cmol_c_ dm^−3^)	2.48	0.34	0.94	2.46	1.53	1.38	1.13	3.57	0.49	0.83	1.44	1.10	1.95	0.97	0.62
EA^5^ (cmol_c_ dm^−3^)	4.62	1.12	3.03	3.28	3.63	1.02	0.78	0.82	1.30	0.83	0.85	0.99	2.19	1.30	0.66
SB^6^ (cmol_c_ dm^−3^)	7.56	1.59	4.57	8.64	5.66	11.48	4.45	14.29	2.88	4.44	9.25	5.08	8.71	4.96	3.23
CEC^7^ (cmol_c_ dm^−3^)	12.18	2.71	7.60	11.92	9.29	12.50	5.23	15.11	4.18	5.27	10.10	6.07	10.90	6.26	3.89
BS^8^ (%)	62.07	58.67	60.13	72.48	60.93	91.88	85.07	94.57	68.88	84.17	91.62	83.74	79.92	79.17	83.01
Silt (g kg^−1^)	166	8	96	208	30	128	128	173	84	43	56	134	133	113	36
Sand (g kg^−1^)	80	904	540	82	732	542	682	420	842	861	664	719	537	817	897
Clay (g kg^−1^)	754	88	364	710	238	330	190	407	74	96	280	147	330	70	67

1 Experimental stations in Brazil: Lon – Londrina; Mar – Maracaí; Rio – Rio verde; Pon – Ponta grossa.

2 Experimental stations in Mozambique: Mur – Muriaze; Nkh – Nkhame; Nte – Ntengo; Rua – Ruace; Sus – Sussundenga.

3 In Mozambique pH was estimated based on the equation pH (CaCl_2_)  = pH (H_2_O) × 0.923 − 0.373 (Ahern et al., 1995).

4 SOM, Soil Organic Matter = 1.724 x soil organic carbon.

5 EA, Exchangeable Acidity  = (Al + H).

6 SB, Sum of Bases  = (K + Ca + Mg).

7 CEC, Cation Exchangeable Capacity  = (EA + SB).

8 BS, Bases Saturation = SB/CEC × 100.

9 na, not available: due to logistic difficulties, rhizobial populations were not estimated in the 2014/2015 crop season in Mozambique.

In Mozambique, all trials were established in areas with no previous soybean cropping history or rhizobial inoculation, whereas in Brazil, the experiments were conducted in areas with or without soybean cultivation history. In Brazil, based on the results of the soil analyses, where applicable, lime was applied to rise bases saturation to 70% (southeast region) or 50% (central region).

### 2.2 Treatments and trials management

Thirty days before sowing, the areas were weeded with 2.5 L ha^−1^ of glyphosate (C_3_H_8_NO_5_P) (in Brazil only). The experiments consisted of the following treatments, (1) NI, non-inoculated and non-N-fertilized control (symbiosis relied on indigenous or naturalized rhizobial populations); (2) NI + N, non-inoculated control with 200 kg of N ha^−1^ as urea (CH_4_N_2_O, 46.6%N), applied 50% at sowing and 50% at R2 (reproductive stage, open flower at one of the two uppermost nodes on the main stem with completely developed leaf; Fehr and Caviness, 1977); (3) SEMIA 5079*,* inoculated with *Bradyrhizobium japonicum* strain SEMIA 5079; (4) SEMIA 5080*,* inoculated with *B. diazoefficiens* strain SEMIA 5080; (5) SEMIA 587*,* inoculated with *B. elkanii* strain SEMIA 587; (6) SEMIA 5019*,* inoculated with *B. elkanii* strain SEMIA 5019; (7) USDA 110*,* inoculated with *B. diazoefficiens* strain USDA 110; (8) 5079 + 5080, inoculated simultaneously with *B. japonicum* strain SEMIA 5079 and *B. diazoefficiens* strain SEMIA 5080 (only in Brazil, as this is the most common combination used in the country). All inoculants were prepared using a peat carrier.

Colony Forming Units (CFU) of each inoculant were verified before sowing to estimate the amount of inoculant that should be applied to release the same number of cells per treatment, of 1.2 × 10^6^ cells seed^−1^. The inoculation was achieved by adding a sucrose solution (10%) to adhere the peat, and mixing seeds and inoculant vigorously and allowing the mixture to dry under the shade for 2 h before sowing. Seeds received no pesticide treatment.

Plot sizes were 6 m × 4 m (in Brazil) or 9 m × 3 m (in Mozambique) and seeds were sown in rows 0.50 m apart to achieve a final population of approximately 300,000 plants ha^−1^ in both countries. The experiments were laid out in randomized complete block design with six (Brazil) or five (Mozambique) replicates. At all experimental sites the plots were separated by 0.50 m-wide lines and 1.5 m-wide terraces to avoid cross contamination with bacteria and/or fertilizer contained in superficial run-off. Sowing dates are shown in Supplementary Table 1 and trials relied on natural rainfall (Supplementary Table 1). Temperatures recorded at sowing during soybean growth stages are shown in Supplementary Table 2.

Immediately before sowing, 300 kg ha^−1^ of fertilizer (0–20–20, N-P-K) were applied in-furrow. In Brazil, at V4 (vegetative stage, four nodes on the main stem with completely unrolled leaves beginning with the unifoliolate nodes; Fehr and Caviness, 1977), plants were sprayed with herbicide, 2.5 L ha^−1^ of C_3_H_8_NO_5_P, and micronutrients, 20 g ha^−1^ of Mo (as Na_2_MoO_4_^.^2H_2_O) and 2.5 g ha^−1^ of Co (as CoCl_2_^.^6H_2_O). In Mozambique, weeding was performed in weekly intervals using manual hoe and, apart from the NI + N treatment, no other fertilizer was added.

### 2.3 Evaluation of nodulation, plant growth, N accumulation, yield and relative effectiveness

Five randomly selected plants were dug out from each plot at V4 (in Brazil) or R3 stages (reproductive stage, pod is 5 mm in length at one of the four uppermost nodes on the main stem with a completely developed leaf; Fehr and Caviness, 1977) (in Mozambique) and taken for assessment of nodulation, plant growth and N accumulation. At the laboratory, plants were cut at the cotyledonary node to separate roots from shoots. Shoots were washed and placed in an air-forced drier at 50 °C for 72 h and weighed to determine shoot dry weight (SDW). Entire shoots were ground (18 mesh) and employed to determine total N accumulation in shoots (TNS) by the salicylate green method (Searle, 1984), with readings taken at the wavelength of 697 nm. Roots and nodules were dried at 50 °C for 72 h. Nodules were then detached from roots, counted, to determine nodule number (NN), before determination of nodule dry weight (NDW).

At physiological maturity, all plants within the central area of 8 m^2^ (in Brazil) or 20 m^2^ (in Mozambique) of each plot were harvested and used to determine the above ground biomass (AGB) (only in Mozambique), grain yield (GY), and grain dry weight (GDW). To determine AGB, plants were cut at the cotyledonary node, dried at 50 °C for 72 h and weighed. For determination of GY, grains were weighed and values adjusted to 13% of moisture content, considering the humidity in a grain moisture tester. One hundred seeds were weighed to determine GDW. Relative effectiveness (RE) was determined as a percentage of SDW of any treatment over that of the NI + N treatment, in the same block (Rufini et al., 2014).

### 2.4 Statistical analysis

Data were checked for normality of errors and homogeneity of variances prior to the statistical analyses. One-way general linear model ANOVA was employed to determine differences among treatments. When significant differences among treatments were detected, Duncan's test was employed to classify the means of the treatments. Differences were considered significant at *p* *≤* 0.10, a level acceptable for strain or inoculant technology recommendation in Brazil (MAPA, 2011). All statistical analyses were performed with software SAS^®^ 9.3 (SAS Institute, North Caroline, USA).

## 3 Results

### 3.1 Soil physical and chemical properties

The experimental sites in Brazil were in four textural classes, Clay, Sandy, Sandy clay loamy and Sandy clay, all of which were represented in Mozambique, apart from Sandy clay (Table 1). In relation to chemical properties, the sites in Mozambique were in relatively more fertile soils, as shown by lower exchangeable acidity and higher base saturation (Table 2).

### 3.2 Indigenous/naturalized rhizobia populations

In Brazil, the population density of naturalized rhizobia varied from ≪ 10 (Maracaí and Rio Verde) to over 10^5^ (Londrina) cells g^−1^ soil (Table 2). In Mozambique, the population sizes of indigenous rhizobia were estimated only in 2013/2014, due to logistic difficulties, and ranged from ≪10 (in Muriaze and Sussundenga) to over 10^3^ cells g^−1^ (Nkhame and Ruace) (Table 2).

### 3.3 Climate and rainfall

Climate type (Table 1), rainfall and temperature data (Supplementary Table 1 and Supplementary Table 2) recorded all through soybean growth stages at the experimental sites are summarized below. In Brazil, the rainfall was particularly low during the transition of soybean from the vegetative to the reproductive growth stages in the 2013/2014 crop season at Londrina and Maracaí. In Mozambique, the rainfall recorded during the transition of soybean from the vegetative to the reproductive growth stages was lower in the 2014/2015 compared to the 2013/2014 crop season at Ntengo, Ruace and Sussundenga.

### 3.4 Nodulation (nodule number and nodule dry weight)

In Brazil, the effect of inoculation on nodulation was observed at Londrina, where all inoculation treatments, except for SEMIA 5080, resulted in increased nodule number (NN) when compared to the non-inoculated control (NI) in the 2013/2014 crop season (Table 3). In 2014/2015, plants inoculated with strains SEMIA 5079 and USDA 110 at Londrina had significantly greater NN and nodule dry weight (NDW) when compared to the NI control. Inoculation with SEMIA 5019 and 5079 + 5080 at Londrina also significantly increased NDW in relation to the NI in 2014/2015, although this was not accompanied by a statistically higher NN. No effects of inoculation on NN and NDW were observed at Maracaí, Rio Verde and Ponta Grossa (Table 3).

**Table 3:** Nodule number (NN, n° plant^−1^), nodule dry weight (NDW, mg plant^−1^), shoot dry weight (SDW, g plant^−1^), total N accumulation in shoots (TNS, mg plant^−1^), grain dry weight (GDW, g 100 seeds^−1^), and relative effectiveness (RE, %) of soybean, cultivars BMX Potência–RR, BRS-359-RR and BRS-360-RR, grown with or without inoculation treatment in the 2013/2014 and 2014/2015 crop seasons at Londrina, Maracaí, Rio Verde and Ponta Grossa, Brazil.

Treatment^1^	Londrina, 2013/2014 crop season - BMX Potência						Londrina, 2014/2015 crop season - BRS-360-RR					
	NN	NDW	SDW	TNS	GDW	RE^2^	NN	NDW	SDW	TNS	GDW	RE^2^
NI	11.8^b^^3^	25.17^a^	0.8^ns^	29.61^ab^	9.7^d^	114.4^ns^	15.8^c^^3^	26.78^bc^	3.2^b^	138.90^bc^	15.6^bc^	95.4^bc^
NI + N	5.6^c^	6.03^b^	0.7	32.12^a^	10.7^a^	100.0^4^	12.4^d^	17.15^c^	3.3^b^	157.35^ab^	16.1^a^	100.0^4^
SEMIA 5079	17.5^a^	31.30^a^	0.6	22.83^bc^	10.1^b^	82.5	22.1^a^	40.76^a^	2.9^b^	126.85^cd^	15.3^c^	89.9^bcd^
SEMIA 5080	15.0^ab^	25.32^a^	0.6	22.49^c^	10.0^bc^	82.9	18.8^b^	34.27^ab^	2.9^b^	126.37^cd^	15.5^c^	88.8^bcd^
SEMIA 587	17.0^a^	29.88^a^	0.6	24.42^bc^	10.0^bc^	87.1	17.0^bc^	28.12^b^	3.0^b^	119.75^cd^	15.7^bc^	88.2^cd^
SEMIA 5019	16.4^a^	28.48^a^	0.6	25.34^bc^	9.8^cd^	89.4	17.2^bc^	44.55^a^	3.4^b^	153.18^ab^	15.3^c^	102.6^b^
USDA 110	17.8^a^	31.91^a^	0.5	19.97^c^	10.1^b^	75.0	18.3^b^	40.17^a^	2.5^c^	108.45^d^	15.4^c^	75.5^d^
5079 + 5080	17.4^a^	33.95^a^	0.7	23.34^bc^	10.0^bc^	95.5	17.5^bc^	40.22^a^	3.9^a^	175.01^a^	15.9^ab^	118.9^a^
*p -* value	0.00	0.00	0.17	0.04	0.00	0.15	0.00	0.00	0.01	0.00	0.00	0.00
C.V. (%)	24.65	31.52	24.45	25.33	2.15	26.45	11.74	29.93	17.66	15.86	2.06	13.95
	Maracaí, 2013/2014 crop season - BMX Potência						Rio Verde, 2013/2014 crop season - BMX Potência					
NI	15.3^ns^^3^	75.62^ns^	1.3^d^	33.27^b^	13.3^a^	86.7^d^	27.4^ns^^3^	107.65^a^	2.5^ns^	69.36^b^	13.0^a^	133.5^a^
NI + N	13.3	69.38	1.6^bc^	51.27^a^	13.0^b^	100.0^4^	20.8	40.09^b^	2.4	92.81^a^	12.9^ab^	100.0^4^
SEMIA 5079	13.5	80.52	1.6^abc^	44.57^a^	13.3^a^	112.5^bc^	26.0	103.53^a^	2.2	70.10^b^	13.1^a^	114.9^abc^
SEMIA 5080	17.5	78.77	1.7^ab^	48.53^a^	12.9^c^	117.1^ab^	26.6	97.06^a^	2.5	69.47^b^	12.7^bc^	127.8^ab^
SEMIA 587	12.2	86.56	1.7^ab^	47.67^a^	13.0^b^	123.4^ab^	23.1	91.05^a^	2.0	61.80^bc^	12.9^ab^	106.9^c^
SEMIA 5019	15.2	72.05	1.4^cd^	38.15^b^	12.9^c^	96.2^cd^	24.6	90.71^a^	2.0	59.03^c^	12.7^c^	112.1^bc^
USDA 110	9.1	68.73	1.3^d^	36.50^b^	12.8^c^	86.7^d^	23.6	91.04^a^	2.0	58.22^c^	13.1^a^	107.5^c^
5079 + 5080	13.9	64.83	1.9^a^	50.42^a^	13.4^a^	132.5^a^	27.6	110.54^a^	2.3	68.39^b^	13.1^a^	129.2^ab^
*p -* value	0.11	0.66	0.00	0.00	0.00	0.00	0.28	0.00	0.23	0.00	0.02	0.08
C.V. (%)	32.54	27.93	15.15	14.69	1.12	17.30	20.56	23.33	19.72	13.06	1.92	15.64
	Ponta Grossa, 2014/2015 crop season – BRS-359-RR											
NI	115.1^ns^^3^	402.89^ns^	5.3^ns^	196.85^bc^	12.7^c^	67.3^c^						
NI + N	93.2	341.69	7.4	312.67^a^	13.5^a^	100.0^4^						
SEMIA 5079	111.0	447.73	6.1	215.83^bc^	13.1^ab^	72.9^bc^						
SEMIA 5080	103.4	365.31	5.4	180.88^c^	13.1^ab^	69.1^bc^						
SEMIA 587	111.1	469.49	6.0	248.73^b^	13.1^ab^	71.3^bc^						
SEMIA 5019	130.4	421.40	6.5	237.31^bc^	12.9^bc^	81.2^ab^						
USDA 110	96.7	356.11	6.4	245.77^b^	12.9^bc^	87.9^a^						
5079 + 5080	107.8	392.09	7.4	250.62^b^	12.9^bc^	90.0^a^						
*p -* value	0.20	0.11	0.22	0.02	0.04	0.01						
C.V. (%)	21.29	20.23	25.58	24.81	1.79	15.93						

1 NI*,* non-inoculated control with no N-fertilizer; NI + N*,* non-inoculated control with 200 kg of N ha^−1^, split twice, applied at sowing and R2; SEMIA 5079*,* inoculated with *B. japonicum* strain SEMIA 5079; SEMIA 5080*,* inoculated with *B. diazoefficiens* strain SEMIA 5080; SEMIA 587*,* inoculated with *B. elkanii* strain SEMIA 587; SEMIA 5019*,* inoculated with *B. elkanii* strain SEMIA 5019; USDA 110*,* inoculated with *B. diazoefficiens* strain USDA 110; 5079 + 5080*,* inoculated with *B. japonicum* strain SEMIA 5079 and *B. diazoefficiens* strain SEMIA 5080; All rhizobia were applied at the rate of 1.2 × 10^6^ cells seed^−1^.

2 Determined as a ratio between the SDW of a given treatment and that of the treatment NI + N (Rufini et al., 2014).

3 Means of six replicates and when followed by same letter in the same column are not statistically different (*p* *≤* 0.10, Duncan test).

Strong responses to inoculation were observed at all sites in Mozambique. Plots treated with strains SEMIA 5079, 5080, and 5019 at Muriaze (Table 4) had significantly higher NN and NDW in relation to the NI control in 2013/2014 and 2014/2015. Inoculation with strain SEMIA 5019 at Nkhame (Table 4) in the 2014/2015 crop season, and at Ntengo (Table 5) in both crop seasons also resulted in increased NN and NDW in relation to the NI treatment. Strain SEMIA 587 improved both NN and NDW at Muriaze (Table 4), Ruace (Table 5) and Sussundenga (Table 5) in the 2014/2015 crop season. At Sussundenga (Table 5), the responses were similar to those observed at Muriaze (Table 4).

**Table 4:** Nodule number (NN, n° plant^−1^), nodule dry weight (NDW, mg plant^−1^), shoot dry weight (SDW, g plant^−1^), ground biomass (AGB, kg ha^−1^), grain dry weight (GDW, g 100 seeds^−1^), and relative effectiveness (RE, %) of soybean, cultivar Storm, grown with or without inoculation treatment in the 2013/2014 and 2014/2015 crop seasons at Muriaze and Nkhame, Mozambique.

Treatment^1^	Muriaze, 2013/2014 crop season						Muriaze, 2014/2015 crop season					
	NN	NDW	SDW	AGB	GDW	RE^2^	NN	NDW	SDW	AGB	GDW	RE^2^
NI	6.2^d^^3^	27.40^c^	12.9^c^	5506^ns^	15.6 ^ns^	68.5^ns^	18.5^f^^3^	150.15^e^	22.5^ns^	3199^b^	15.8^ab^	100.8^ns^
NI + N	3.4^e^	27.60^c^	19.8^a^	5610	16.4	100.0^4^	13.5^g^	73.70^fg^	23.5	1955^c^	15.9^ab^	100.0^4^
SEMIA 5079	11.3^c^	123.80^b^	11.9^c^	5402	15.9	66.3	27.1^e^	272.11^d^	18.1	5346^a^	14.1^c^	81.3
SEMIA 5080	14.9^b^	108.72^b^	11.8^c^	5651	16.8	62.1	30.2^d^	419.40^b^	20.7	3986^b^	15.2^bc^	94.2
SEMIA 587	7.2^d^	38.52^c^	12.6^c^	5181	16.6	69.8	33.8^c^	343.96^c^	18.6	3539^b^	14.5^bc^	86.1
SEMIA 5019	23.7^a^	181.73^a^	18.8^ab^	5373	17.5	87.6	46.1^a^	508.70^a^	18.4	4203^b^	14.9^bc^	81.8
USDA 110	7.4^d^	37.64^c^	15.0^bc^	5575	15.8	76.9	35.4^b^	89.20^f^	21.4	3742^b^	14.8^bc^	94.4
*p -* value	0.00	0.00	0.00	0.35	0.21	0.22	0.00	0.00	0.25	0.00	0.06	0.38
C.V. (%)	13.97	29.74	25.50	8.54	6.97	23.00	2.89	11.35	21.29	22.72	8.72	18.80
	Nkhame, 2013/2014 crop season						Nkhame, 2014/2015 crop season					
NI	24.8^a^^3^	29.50^cd^	36.5^ns^	6995^ns^	15.6^bcd^	66.0 ^ns^	8.9^d^^3^	91.63^c^	17.2^e^	3810^ns^	14.8^d^	59.6^d^
NI + N	16.7^cd^	16.95^d^	58.6	8032	17.3^a^	100.0^4^	9.6^d^	68.93^d^	31.3^b^	4797	15.7^ab^	100.0^4^
SEMIA 5079	22.6^ab^	18.65^d^	50.2	7111	17.0^a^	92.1	22.1^b^	93.58^c^	37.8^a^	4223	16.0^a^	130.8^a^
SEMIA 5080	17.7^bcd^	15.96^d^	43.4	7772	15.7^bcd^	79.5	16.4^c^	99.74^c^	21.3^de^	4343	15.0^cd^	70.7^cd^
SEMIA 587	17.3^cd^	42.20^c^	54.0	7131	15.0^d^	94.9	10.0^d^	122.63^b^	36.8^a^	4961	15.4^abc^	124.3^a^
SEMIA 5019	22.5^ab^	90.01^a^	62.7	7209	16.0^bc^	100.5	33.4^a^	156.57^a^	16.8^e^	4621	15.1^cd^	57.7^d^
USDA 110	20.4^abc^	38.40^c^	52.4	7463	16.1^b^	93.2	16.4^c^	52.00^de^	24.3^cd^	4913	15.5^abc^	81.7^c^
*p-*value	0.00	0.00	0.35	0.61	0.00	0.24	0.00	0.00	0.00	0.42	0.02	0.00
C.V. (%)	21.92	31.21	28.83	13.18	4.94	24.96	31.23	20.19	18.41	19.31	3.29	17.38

^ns^ not statistically different (*p* ≤ 1.0, Duncan test).

1 NI*,* non-inoculated control with no N-fertilizer; NI + N*,* non-inoculated control with 200 kg of N ha^−1^, split twice, applied at sowing and R2; SEMIA 5079*,* inoculated with *B. japonicum* strain SEMIA 5079; SEMIA 5080*,* inoculated with *B. diazoefficiens* strain SEMIA 5080; SEMIA 587*,* inoculated with *B. elkanii* strain SEMIA 587; SEMIA 5019*,* inoculated with *B. elkanii* strain SEMIA 5019; USDA 110*,* inoculated with *B. diazoefficiens* strain USDA 110; All rhizobia were applied at the rate of 1.2 × 10^6^ cells seed^−1^.

2 Determined as a ratio between the SDW of a give treatment and that of the treatment NI + N (Rufini et al., 2014).

3 Means of five replicates and when followed by same letter in the same column are not statistically different (*p* ≤ 0.10, Duncan test).

4 Not included in the statistical analysis.

**Table 5:** Nodule number (NN, n° plant^−1^), nodule dry weight (NDW, mg plant^−1^), shoot dry weight (SDW, g plant^−1^), above ground biomass (AGB, kg ha^−1^), grain dry weight (GDW, g 100 seeds^−1^), and relative effectiveness (RE, %) of soybean, cultivar Storm, grown with or without inoculation treatment in the 2013/2014 and 2014/2015 crop seasons at Ntengo, Ruace and Sussundenga, Mozambique.

Treatment^1^	Ntengo, 2013/2014 crop season						Ntengo, 2014/2015 crop Season					
	NN	NDW	SDW	AGB	GDW	RE^2^	NN	NDW	SDW	AGB	GDW	RE^2^
NI	6.0^f^^3^	96.40^b^	19.1^d^	6081^ns^	14.9^ns^	80.3^b^	29.0^b^^3^	77.90^d^	18.8^ns^	2740^ns^	16.2^ns^	102.1^ns^
NI + N	6.8^ef^	90.85^b^	24.0^ab^	5265	15.8	100.0^4^	18.0^c^	44.70^e^	18.6	3543	16.4	100.0^4^
SEMIA 5079	9.0^cd^	128.15^b^	19.5^cd^	6423	15.5	82.8^b^	39.9^a^	282.21^a^	21.8	3167	16.0	118.2
SEMIA 5080	22.2^a^	181.00^a^	20.0^cd^	5581	15.2	84.2^b^	28.6^bc^	92.38^d^	16.2	3202	16.3	88.0
SEMIA 587	10.6^c^	127.35^b^	26.5^a^	5790	16.1	111.7^a^	29.5^b^	125.45^c^	19.8	3475	15.9	108.1
SEMIA 5019	13.4^b^	201.56^a^	19.7^cd^	5505	15.9	81.9^b^	42.7^a^	195.38^b^	18.3	3219	15.7	99.3
USDA 110	8.2^de^	118.84^b^	22.4^bc^	5395	15.8	93.5^b^	22.0^bc^	80.49^d^	16.8	3403	16.0	90.7
*p -* value	0.00	0.00	0.00	0.19	0.25	0.00	0.00	0.00	0.77	0.46	0.26	0.74
C.V. (%)	14.90	23.42	12.41	12.19	5.57	13.94	31.18	20.22	28.14	17.30	3.94	29.82
	Ruace, 2013/2014 crop season						Ruace, 2014/2015 crop season					
NI	9.9^e^^3^	100.00^e^	26.8^b^	8806^ns^	17.5^e^	81.7^a^	7.4^d^^3^	48.68^de^	7.8^cd^	2267^ns^	14.2^ns^	54.9^bcd^
NI + N	2.4 ^f^	17.64^f^	32.8^a^	9167	18.3^d^	100.0^4^	6.2^d^	29.24^e^	15.0^a^	2526	15.6	100.0^4^
SEMIA 5079	21.6^c^	296.85^b^	28.0^b^	8791	18.8^cd^	86.1^a^	22.3^b^	151.67^b^	15.0^a^	2766	14.8	106.3^a^
SEMIA 5080	41.1^a^	426.48^a^	26.2^b^	9178	19.4^bc^	80.1^a^	34.5^a^	198.28^a^	17.4^a^	3156	16.1	119.8^a^
SEMIA 587	23.5^c^	217.60^c^	25.2^b^	8920	19.6^b^	77.6^a^	25.1^b^	124.12^c^	10.4^b^	2497	15.0	72.5^b^
SEMIA 5019	37.9^b^	409.28^a^	26.2^b^	8531	20.2^a^	80.4^a^	12.3^c^	67.12^d^	7.2^cd^	2799	14.7	48.5^cd^
USDA 110	9.9^e^	139.92^d^	22.0^c^	8304	19.2^bc^	67.5^b^	11.7^c^	37.80^e^	5.6^d^	2644	14.8	38.2^d^
*p -* value	0.00	0.00	0.00	0.16	0.00	0.02	0.02	0.00	0.00	0.35	0.34	0.00
C.V. (%)	12.31	15.88	9.53	7.48	2.82	9.47	9.47	23.72	20.80	22.61	8.35	23.91
	Sussundenga, 2013/2014 crop season						Sussundenga, 2014/2015 crop season					
NI	8.9^e^^3^	118.98^ef^	8.2^c^	6056^cde^	9.1^ns^	35.8^b^	5.5^d^^3^	65.80^fg^	11.4^c^	5519^ns^	13.5^ns^	138.5^bc^
NI + N	4.1^f^	64.30^f^	23.3^a^	6728^bcd^	9.5	100.0^4^	5.8^d^	53.04^g^	8.0^d^	6512	15.3	100.0^4^
SEMIA 5079	20.8^c^	334.72^c^	17.0^b^	7250^a−d^	9.3	73.3^a^	15.5^c^	129.80^d^	10.8^c^	5836	14.0	128.8^c^
SEMIA 5080	30.0^b^	439.03^b^	16.9^b^	7867^ab^	9.4	71.5^a^	34.0^ab^	188.10^c^	15.4^a^	6912	13.9	163.6^a^
SEMIA 587	15.4^d^	176.35^de^	8.9^c^	5861^de^	9.2	38.6^b^	32.2^b^	276.30^b^	12.8^abc^	6022	14.2	138.2^bc^
SEMIA 5019	39.5^a^	594.65^a^	17.0^b^	8528^a^	9.5	72.3^a^	35.4^a^	351.48^a^	14.8^ab^	6482	15.9	160.9^ab^
USDA 110	18.4^cd^	246.38^cd^	10.8^c^	7437^abc^	9.1	47.3^b^	5.7^d^	85.41^ef^	12.5^bc^	6298	15.3	141.6^abc^
*p-*value	0.00	0.00	0.00	0.00	0.84	0.00	0.00	0.00	0.00	0.39	0.13	0.02
C.V. (%)	20.65	28.76	25.13	17.50	6.23	28.78	15.66	12.04	19.36	17.20	9.70	14.11

^ns^ not statistically different (*p* ≤ 0.10, Duncan test).

1 NI*,* non-inoculated control with no N-fertilizer; NI + N*,* non-inoculated control with 200 kg of N ha^−1^, split twice, applied at sowing and R2; SEMIA 5079*,* inoculated with *B. japonicum* strain SEMIA 5079; SEMIA 5080*,* inoculated with *B. diazoefficiens* strain SEMIA 5080; SEMIA 587*,* inoculated with *B. elkanii* strain SEMIA 587; SEMIA 5019*,* inoculated with *B. elkanii* strain SEMIA 5019; USDA 110*,* inoculated with *B. diazoefficiens* strain USDA 110; All rhizobia were applied at the rate of 1.2 × 10^6^ cells seed^−1^.

2 Determined as a ratio between the SDW of a given treatment and that of the treatment NI + N (Rufini et al., 2014).

3 Means of five replicates and when followed by same letter in the same column are not statistically different (*p* ≤ 0.10, Duncan test).

4 Not included in the statistical analysis.

Although the use of N-fertilizer decreased nodulation in Brazil, as indicated by a significant reduction of NN and/or NDW at Londrina and Rio Verde (Table 3), the detrimental effects of N-fertilizer application on nodulation were more evident in Mozambique, where significant reduction on NN and/or NDW was observed at Muriaze, Nkhame (Table 4), Ntengo and Ruace (Table 5).

### 3.5 Plant growth and nitrogen accumulation

In Brazil, strains SEMIA 587, 5079, and 5080, and the combination 5079 + 5080 significantly improved shoot dry weight (SDW) and total N accumulated in shoots (TNS) when compared to the non-inoculated (NI) control at Maracaí (Table 3). The combination 5079 + 5080 also resulted in statistically higher SDW and TNS at Londrina (2014/2015) than the NI treatment (Table 3).

In Mozambique, inoculants carrying strains USDA 110, SEMIA 5079 and 587 at Nkhame (2014/2015) (Table 4), SEMIA 587 and USDA 110 at Ntengo (2013/2014) (Table 5), SEMIA 5079, 5080 and 587 at Ruace (2014/2015) (Table 5) and SEMIA 5080 and 5019 at Sussundenga (both seasons) (Table 5) had higher SDW than the NI treatment.

### 3.6 Above ground biomass at harvest, grain yield and grain dry weight

**Fig. 1:**
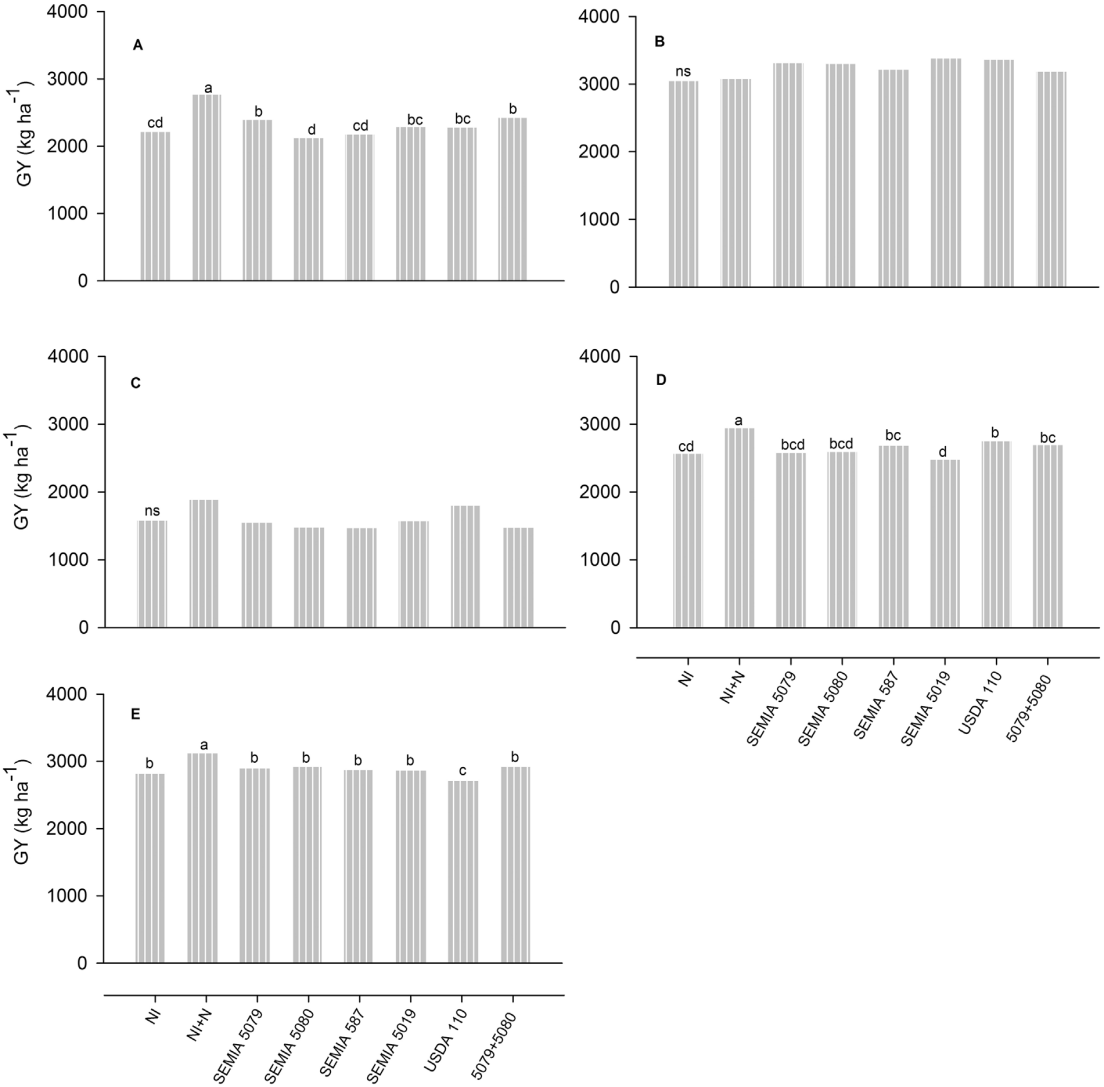
Grain yield (GY, kg ha^−1^) of soybean grown with or without inoculation treatment in Brazil at Londrina in the 2013/2014 (A) and 2014/2015 (B) crop seasons, Maracaí in the 2013/2014 crop season (C), Rio Verde in the 2013/2014 crop season (D) and Ponta Grossa in the 2014/2015 crop season (E). Three soybean varieties, BMX Potência–RR (A, C and D), BRS 360–RR (B) and BRS-359–RR (E), were employed in the trials. NI*,* non-inoculated control with no N-fertilizer; NI + N*,* non-inoculated control with 200 kg of N ha^−1^, split twice, applied at sowing and R2; SEMIA 5079*,* inoculated with *B. japonicum* strain SEMIA 5079; SEMIA 5080*,* inoculated with *B. diazoefficiens* strain SEMIA 5080; SEMIA 587*,* inoculated with *B. elkanii* strain SEMIA 587; SEMIA 5019*,* inoculated with *B. elkanii* strain SEMIA 5019; USDA 110*,* inoculated with *B. diazoefficiens* strain USDA 110; 5079 + 5080*,* inoculated with *B. japonicum* strain SEMIA 5079 and *B. diazoefficiens* strain SEMIA 5080; All rhizobia were applied at the rate of 1.2 × 10^6^ cells seed^−1^. Bars are means of six replicates and when followed by same letter in the same graph are not statistically different (*p* *≤* 0.10, Duncan test); ns – not significantly different (*p* *≤* 0.10, Duncan test).

The effect of inoculation on grain yield (GY) was observed at two sites in Brazil. Compared to the non-inoculated control, treatments SEMIA 5079 + 5080 and SEMIA 5079 significantly increased GY at Londrina (2013/2014), while USDA 110 improved GY at Rio Verde (Fig. 1). Strain USDA 110 was the best performing strain across sites and crop seasons with grain yield gains of 5% in relation to the non-inoculated (NI) control (Supplementary Table 3). GY gains attributable to N-fertilizer varied from 11% at Ponta Grossa to 25% at Londrina (2013/2014 crop season) (Fig. 1). The average N-fertilizer gain on GY across sites and crop seasons was 11% in relation to the NI treatment, compared to 5% of USDA 110 (Supplementary Table 3).

In Brazil, plots treated with strains SEMIA 5079, 5080 and 587 had significantly higher grain dry weight (GDW) compared to the non-inoculated control at Londrina (2013/2014) and Ponta Grossa (Table 3). Remarkable inoculation effects on above ground biomass and yield components were observed in Mozambique. Plots treated with strains SEMIA 5079 at Muriaze (2014/2015) (Table 4), SEMIA 5080 and SEMIA 5019 at Sussundenga (2013/2014) (Table 5) had higher above ground biomass (AGB) than the non-inoculated control. Analysis across sites revealed that in the 2013/2014 crop season plants treated with strain SEMIA 5080 had the best and significantly higher (8%) AGB than the NI control plants (Supplementary Table 4). In the 2014/2015 crop season all strains resulted in higher (16–23%) and significant AGB gains relatively to the non-inoculated control, and strains SEMIA 5080, 5019 and USDA 110 resulted in significantly higher AGB gains of 12, 10 and 9%, respectively, in relation to N-fertilized control (Supplementary Table 4).

**Fig. 2:**
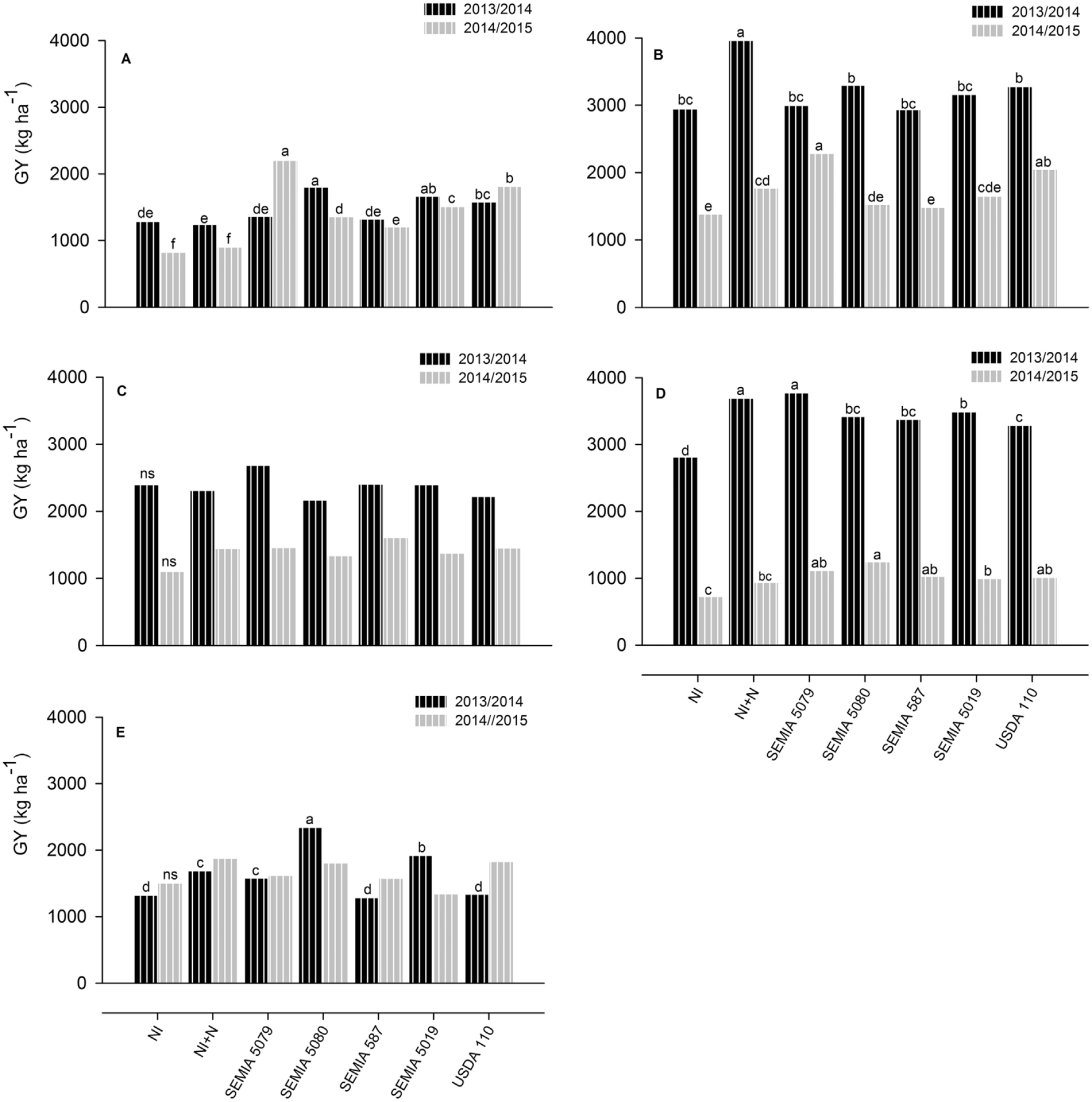
Grain yield (GY, kg ha^−1^) of soybean, cultivar Storm, grown with or without inoculation treatment in Mozambique at Muriaze (A), Nkhame (B), Ntengo (C), Ruace (D) and Sussundenga (E) in the 2013/2014 and 2014/2015 crop seasons. NI*,* non-inoculated control with no N-fertilizer; NI + N*,* non-inoculated control with 200 kg of N ha^−1^, split twice, applied at sowing and R2; SEMIA 5079*,* inoculated with *B. japonicum* strain SEMIA 5079; SEMIA 5080*,* inoculated with *B. diazoefficiens* strain SEMIA 5080; SEMIA 587*,* inoculated with *B. elkanii* strain SEMIA 587; SEMIA 5019*,* inoculated with *B. elkanii* strain SEMIA 5019; USDA 110*,* inoculated with *B. diazoefficiens* strain USDA 110; All rhizobia were applied at the rate of 1.2 × 10^6^ cells seed^−1^. Bars are means of five replicates and when followed by same letter in the same location and crop season are not statistically different (*p* *≤* 0.10, Duncan test); ns – not significantly different (*p* *≤* 0.10, Duncan test).

In the 2013/2014 crop season, inoculants with strains SEMIA 5080, 5019 and USDA 110 at Muriaze, all strains at Ruace, and strains SEMIA 5079, 5080 and 5019 at Sussundenga significantly increased GY in relation to the non-inoculated control (Fig. 2). In the following crop season, all inoculated plants at Muriaze and Ruace significantly improved GY compared to the non-inoculated ones. Inoculation with strains SEMIA 5079 and USDA 110 at Nkhame also resulted in increased GY in relation to the non-inoculated treatment in the 2014/2015 crop season (Fig. 2). All inoculated plants had significantly higher GY than the non-inoculated ones across experimental sites in both 2013/2014 (GY gains range 5–21%) and 2014/2015 (24–57%) crop seasons (Supplementary Table 4). In the 2014/2015 crop season, inoculation with SEMIA 5079 and USDA 110 resulted in significant GY gains of 31 and 23%, respectively, in relation to the N-fertilized treatment (Supplementary Table 4). SEMIA 5079, 5080, 5019 and USDA 110 were the best strains across experimental sites and crop seasons with grain yield gains of 20–29% over the non-inoculated control, a similar or better performance than the 20% yield gains obtained with the NI + N control (Supplementary Table 4).

Inoculation with strains SEMIA 5079, SEMIA 587 and USDA 110 at Nkhame (2014/2015) (Table 4), and all strains in 2013/2014 at Ruace (Table 5) resulted in significant increased grain dry weight (GDW) compared to the non-inoculated control. N-fertilizer application significantly improved GDW compared to the non-inoculatd control at Nkhame (Table 4) and Ruace (2013/2014 crop season) (Table 5). Interestingly, in the 2013/2014 crop season, N-fertilizer treatment was outperformed by treatments with strains SEMIA 5080, 587 and 5019 and USDA 110 at Ruace (Table 5).

### 3.7 Relative effectiveness

Plants inoculated with 5079 + 5080 had significantly higher relative effectiveness (RE) compared to those that relied on naturalized rhizobia at Londrina (2014/2015), Maracaí and Ponta Grossa (Table 3). Inoculation with strains SEMIA 5079, 5080 and 587 at Maracaí, SEMIA 5019 and USDA 110 at Ponta Grossa, also resulted in increased RE in relation to the non-inoculated treatment (Table 3).

In Mozambique, plants treated with strains SEMIA 587 at Ntengo and SEMIA 5079, 5080 and 5019 at Sussundenga (Table 5) had significantly greater RE than the non-inoculated control in 2013/2014. In 2014/2015, inoculation with strains SEMIA 5079 and 587 and USDA 110 at Nkhame (Table 4), SEMIA 5079 and 5080 at Ruace (Table 5) and SEMIA 5080 at Sussundenga (Table 5) significantly increased RE in relation to the NI treatment.

## 4 Discussion

Brazilian soils are originally devoid of rhizobia capable of nodulating soybean, but strain selection programs started early with soybean expansion in the 1960s (Hungria et al., 2006a, Hungria and Mendes, 2015). Elite inoculant strains from Australia and the USA were field tested in Brazil to verify their adaptability to the local agro-climatic conditions, N_2_-fixation effectiveness and ability to compete for nodule occupancy (Hungria and Mendes, 2015). Following years of extensive trials and research improvements, four strains, *B. elkanii* SEMIA 587 and SEMIA 5019, *B. japonicum* SEMIA 5079 and *B. diazoefficiens* SEMIA 5080 are currently employed in commercial inoculants for the crop in Brazil, in single or double-strain combinations (Hungria et al., 2005, Hungria et al., 2013, Campo et al., 2009). The double-strain inoculant SEMIA 5079 + 5080 represents over 80% of the commercial inoculants sold in the country and is the farmers’ choice in the Cerrados region (Hungria et al., 2006a, Hungria and Mendes, 2015), an edaphic type of savannah. Yield enhancements of 4–12% attributable to the inoculant combination SEMIA 5079 + 5080 have been reported in bradyrhizobia populated soils (Vargas and Hungria, 2000, Campo et al., 2009).

The superiority of the combination SEMIA 5079 + 5080 was confirmed in our study, where it consistently resulted in the highest nodulation, plant growth, N accumulation in shoots, grain dry weight and symbiotic effectiveness (Table 3, Supplementary Table 3). This combination of strains resulted in grain yield gains over the non-inoculated control of 9 and 5%, respectively, in the 2013/2014 and 2014/2015 crop seasons at Londrina (Fig. 1, Supplementary Table 3), the site with the highest naturalized rhizobial population, estimated at 2 × 10^5^ cells g^−1^ of soil (Table 2). These yield gains are within the 3.2–14.5% interval of re-inoculation yield benefit reported in Brazil (Mendes et al., 2004, Hungria et al., 2006a, Hungria et al., 2013). Despite the better performance of double over single-strain inoculants reported here, in countries like Mozambique, where soybean is a relatively new crop, it is much easier to introduce the concept of single-strain inoculants. This concept is currently being revisited in Brazil. We should also mention that, in general, in the 2013/2014 crop season the yields recorded at the experimental sites in Brazil were lower than those recorded in the previous crop seasons. The yield decreases can be ascribed to the lack of adequate rainfall during R3 reproductive stage (Supplementary Table 1), in which short rainfall records substantially reduce grain yields.

Interestingly, USDA 110, a strain that has never been used in commercial inoculants in Brazil, was among the best performing strains even at Londrina (Fig. 1, Table 3). This is in agreement with reports of outstanding competitiveness (George et al., 1987, Abaidoo and van Kessel, 1989, Abaidoo et al., 1990, McDermott and Graham, 1990, Thies et al., 1992) and N_2_-fixation effectiveness (Abaidoo et al., 2007, Agoyi et al., 2016) of this strain.

In Mozambique, where three out of the five surveyed fields had ≪ 100 cells g^−1^ of soil (Table 2), inoculation responses were much stronger, as indicated by average yield gains over the non-inoculated control of 5–57% (Fig. 2, Supplementary Table 4). Despite a general positive response to inoculation, particularities were observed at each site. Grain yield gains were far greater at Ruace (17–34%) than Nkhame (2–12%) in the 2013/2014 crop season, although both sites had similar rhizobial population size, of 1 × 10^3^ cells g^−1^ of soil. We may thus suppose that an appreciable proportion of the rhizobial population present at Ruace is composed of ineffective bacteria (Osunde et al., 2003).

In Mozambique, relatively better grain yields were recorded in the first compared to the second crop season at Muriaze, Nkhame, Ntengo and Ruace (Fig. 2). The lower rainfall recorded during stage R3 at the four experimental sites in the 2014/2015 compared to the 2013/2014 crop season (Supplementary Table 1) may have contributed to the decrease in grain yield from the first to the second crop season.

Intriguingly, grain yield and grain dry weight improved from the first to the second crop season at Sussundenga (Fig. 2, Table 5, respectively) despite considerably better environmental conditions recorded in the first compared to the second crop season. The rainfall amount and distribution was more favorable in the first crop season (Supplementary Table 1), while the temperatures recorded in both crop seasons were similar and within the suitable range (20–30 °C) for soybean growth (Supplementary Table 2). Grain dry weight is the yield component known to reduce remarkably under drought stress occurring during R5 (Dornbos and Mullen, 1991). In this study, however, the slightly lower rainfall and higher temperatures recorded during and/or just after R5 in the first season are unlikely to have caused enough evapotranspiration rates to explain the grain dry weight and grain yield differences. Interestingly, the above ground biomass was much higher in the first compared to the second crop season (Table 5), agreeing with the better environmental conditions recorded in the first crop season.

Soybean inoculation success in Brazil can be explained by the elite strains used and, in the case of re-inoculation, the improvement of nodulation of the crown root by the inoculant strains, even in soils with naturalized populations. Inoculant strains typically dominate occupancy of crown root nodules (McDermott and Graham, 1989, Graham, 2008) but are unable to sustain high population levels all through the growing root system (Madsen and Alexander, 1982, McDermott and Graham, 1989, Wadisirisuk et al., 1989). The inability of inoculant strains to fully explore the root profile allows positional advantage to be taken by the strains already in the soil on the competition for lateral root infections sites (Vlassak et al., 1997; López-García et al., 2002; Bogino et al., 2008). Furthermore, crown root nodules usually undergo a senescence process around R4 reproductive stage (pod 2 cm in length and one of the two four uppermost nodes on the main stem with completely developed leaf; Fehr and Caviness, 1977) (Bergersen, 1958, Espinosa-Victoria et al., 2000, Alesandrini et al., 2003) just before N_2_-fixation reaches maximum levels (Thibodeau and Jaworski, 1975). This means that symbiosis will markedly be influenced by the symbiotic effectiveness of naturalized rhizobia. It is, therefore, possible that the observed re-inoculation responses represent a combined effect of the N_2_ fixed in the crown and lateral nodules predominately occupied by inoculant and naturalized strains, respectively (López-García et al., 2002; Bogino et al., 2008; Graham, 2008). In annually cropped soybean areas, inoculated soybean plants frequently exhibit profuse nodulation on the crown root, contrasting with delayed infections occurring at 1–2 cm below the crown on control plots (Hungria and Mendes, 2015), which elucidates the positional difference of inoculant and naturalized strains in the root profile.

N-fertilizer reduced nodule number and dry weight in both countries, supporting previous observations that increased levels of mineral N in the rhizosphere inhibit soybean nodule formation and functioning (Arrese-Igor et al., 1997, Hungria et al., 2006b, Hungria and Mendes, 2015). Moreover, in Mozambique, inoculation with strains SEMIA 5079 and USDA 110, the best performing strains across sites in the 2014/2015 crop season, resulted in significant grain yield gains, of 31 and 23%, respectively, in relation to the N-fertilized control (Fig. 2, Supplementary Table 4). This corroborates previous evidence of the profitably of inoculation compared to N-fertilizer application (Hungria et al., 2006a, Hungria and Mendes, 2015). In Brazil, however, N-fertilizers increased grain yield in three out of the five experiments. The low rainfall recorded at the experimental sites, particularly during R3 (Supplementary Table 1) may explain the low yields. In addition, it is broadly reported that under water stressing conditions BNF is more affected than the assimilation of mineral N (Serraj et al., 2001, Dwivedi et al., 2015). Despite the observed yield gains, N-fertilizer application would not be profitable, considering the typically high fertilizer prices in the Brazilian market. However, concerns are raised in Brazil that the increasing periods of water stress, due to the global climatic changes, might lead to the need of application of N fertilizers, with serious economic and environmental impacts. On the contrary, in Mozambique the use of N-fertilizer did not provide better results than those obtained with the best performing strains, SEMIA 5079, SEMIA 5080, SEMIA 5019 and USDA 110, considering averages across sites and crop seasons (Fig. 2, Supplementary Table 4).

In conclusion, elite strains either selected in Brazil or in USA improved soybean growth, yield and grain dry weight in Brazil and Mozambique. The best treatments across experimental sites in Brazil were SEMIA 5079 + 5080, SEMIA 5079 and USDA 110, with average grain yield gains of 4–5%. In Mozambique, the best treatments were SEMIA 5079, SEMIA 5080, SEMIA 5019 and USDA 110, with overall grain yield gains of 20–29%. These results suggest that the strains SEMIA 5079, SEMIA 5080 and USDA 110 hold the best potential as commercial inoculants in both countries. Strains SEMIA 5079 and SEMIA 5080 have shown to be very effective in fixing nitrogen and tolerant to the harsh conditions of the Brazilian Cerrados (Hungria and Mendes, 2015). USDA 110 is also very effective (Abaidoo et al., 2007, Agoyi et al., 2016) and competitive (George et al., 1987, McDermott and Graham, 1990). Therefore, these strains are likely to adapt well not only in Brazil and Mozambique, but also in other countries with similar agro-climatic conditions. The feasibility of transference of soybean inoculation technologies between countries with relatively similar agro-climatic conditions can save time, labor and money, and speed up the introduction of productive and sustainable cropping systems, as is the case of the soybean in Africa.
