# Supplementary material for: Feasibility of transference of inoculation-related technologies: A case study of evaluation of soybean rhizobial strains under the agro-climatic conditions of Brazil and Mozambique
Source: Agric Ecosyst Environ. 2018 Jul 1;261:230–40. doi: 10.1016/j.agee.2017.06.037 (PMC5946691; doi:10.1016/j.agee.2017.06.037)
Supplement: Supplementary file 1 [file mmc1.doc]

**Feasibility of transference of inoculation-related technologies: A case study of evaluation of soybean rhizobial strains under the agro-climatic conditions of Brazil and Mozambique**

Amaral Machaculeha Chibeba, Stephen Kyei-Boahen, Maria de Fátima Guimarães, Marco Antonio Nogueira, Mariangela Hungria

**Supplementary Material**

**Supplementary Table 1**

Sowing dates and rainfall recorded during soybean growth at the experimental sites in the 2013/2014 and 2014/2015 crop seasons.

| Experimental  site | Sowing  date | | Rainfall (mm) recorded in different soybean growth stages^1^ | | | | | | | | | | | | | | |
| --- | --- | --- | --- | --- | --- | --- | --- | --- | --- | --- | --- | --- | --- | --- | --- | --- | --- |
|  |  |  | VE | VC | V1 | V2 | V3 | V4 | V5 | V6 | R1 | R3 | R4 | R5 | R6 | R7 | R8 |
| Brazil, crop season 2013/2014 | | | | | | | | | | | | | | | | | |
| Londrina | | 24-Oct-13 | 17.7 | 10.3 | 19.1 | 23.5 | 37.0 | 14.9 | 14.5 | 0.0 | 0.0 | 60.2 | 104.3 | 13.0 | 2.5 | 131.0 | 65.3 |
| Maracaí | | 23-Oct-13 | 23.0 | 11.5 | 47.6 | 22.7 | 34.9 | 31.0 | 24.2 | 3.1 | 0.0 | 1.4 | 209.0 | 61.4 | 116.9 | 32.5 | 33.7 |
| Rio Verde | | 06-Nov-13 | 79.2 | 24.0 | 145.2 | 17.6 | 75.6 | 27.4 | 70.0 | 3.8 | 56.4 | 28.8 | 24.8 | 11.6 | 42.6 | 30.6 | 168.0 |
| Mozambique, crop season 2013/2014 | | | | | | | | | | | | | | | | | |
| Muriaze | | 25-Dec-13 | 160.6 | 54.7 | 58.9 | 117.9 | 17.7 | 0.0 | 4.4 | 38.5 | 8.5 | 194.4 | 39.5 | 11.6 | 16.1 | 72.7 | 25.8 |
| Nkhame | | 20-Dec-13 | 64.0 | 104.0 | 28.0 | 55.0 | 5.0 | 53.0 | 30.0 | 56.0 | 3.0 | 89.0 | 47.0 | 7.0 | 9.0 | 81.0 | 7.0 |
| Ntengo | | 17-Dec-13 | 104.2 | 8.9 | 63.1 | 22.5 | 65.2 | 3.9 | 22.3 | 19.4 | 55.5 | 29.5 | 92.9 | 67.0 | 26.2 | 97.3 | 9.4 |
| Ruace | | 20-Dec-13 | 61.8 | 20.1 | 56.6 | 111.7 | 25.7 | 115.7 | 66.8 | 28.1 | 49.0 | 60.7 | 129.4 | 101.0 | 40.6 | 9.1 | 107.1 |
| Sussundenga | | 21-Dec-13 | 259.0 | 67.2 | 68.0 | 24.9 | 10.2 | 109.4 | 344.5 | 20.5 | 210.8 | 208.3 | 127.0 | 208.6 | 32.3 | 59.9 | 31.5 |
| Brazil, crop season 2014/2015 | | | | | | | | | | | | | | | | | |
| Londrina | | 04-Nov-14 | 11.2 | 8.2 | 95.4 | 4.6 | 35.5 | 19.6 | 21.9 | 0.1 | 117.8 | 9.6 | 2.9 | 61.6 | 57.5 | 90.7 | 50.4 |
| Ponta Grossa | | 18-Nov-14 | 88.1 | 0.0 | 27.1 | 66.8 | 2.3 | 112.9 | 27.8 | 39.9 | 8.3 | 66.3 | 14.6 | 103.3 | 118.0 | 90.9 | 29.3 |
| Mozambique, crop season 2014/2015 | | | | | | | | | | | | | | | | | |
| Muriaze | | 05-Jan-15 | 245.5 | 41.6 | 2.3 | 53.9 | 35.9 | 75.1 | 21.2 | 39.5 | 32.9 | 48.2 | 226.1 | 60.9 | 23.1 | 3.5 | 7.4 |
| Nkhame | | 23-Dec-14 | 118.0 | 16.0 | 35.0 | 18.0 | 25.0 | 48.0 | 98.0 | 62.0 | 6.0 | 28.0 | 84.0 | 122.0 | 30.0 | 47.0 | 15.0 |
| Ntengo | | 22-Dec-14 | 89.7 | 40.9 | 32.7 | 49.6 | 70.6 | 5.9 | 48.6 | 3.9 | 14.0 | 21.1 | 100.6 | 65.3 | 32.4 | 29.6 | 6.8 |
| Ruace | | 21-Jan-15 | 48.9 | 43.4 | 33.1 | 12.6 | 44.9 | 43.2 | 43.4 | 6.9 | 77.9 | 6.2 | 20.0 | 0.6 | 17.2 | 0.0 | 3.0 |
| Sussundenga | | 29-Dec-14 | 530.6 | 0.2 | 37.5 | 9.1 | 1.2 | 32.2 | 164.0 | 0.0 | 0.0 | 87.6 | 62.7 | 10.9 | 76.9 | 127.7 | 81.5 |

**^1^**As defined by Fehr and Caviness (1977).

**Supplementary Table 2**

Sowing dates and temperature recorded during soybean growth stages at the experimental sites in the 2013/2014 and 2014/2015 crop seasons.

| Experimental  site | Sowing  date^2^ | | Temperature (^o^C) in different soybean growth stages^1^ | | | | | | | | | | | | | | |
| --- | --- | --- | --- | --- | --- | --- | --- | --- | --- | --- | --- | --- | --- | --- | --- | --- | --- |
|  |  |  | VE | VC | V1 | V2 | V3 | V4 | V5 | V6 | R1 | R3 | R4 | R5 | R6 | R7 | R8 |
| Brazil, crop season 2013/2014 | | | | | | | | | | | | | | | | | |
| Londrina | | 24-Oct-13 | 22.7 | 21.5 | 24.6 | 21.6 | 23.3 | 24.9 | 23.0 | 23.3 | 23.2 | 24.3 | 23.7 | 24.0 | 27.3 | 24.1 | 23.3 |
| Rio Verde | | 06-Nov-13 | 23.6 | 24.1 | 22.6 | 23.9 | 24.4 | 23.8 | 23.2 | 24.3 | 22.6 | 23.6 | 24.0 | 23.2 | 22.9 | 24.3 | 22.4 |
| Mozambique, crop season 2013/2014 | | | | | | | | | | | | | | | | | |
| Muriaze | | 25-Dec-13 | 26.8 | 26.1 | 26.7 | 25.6 | 26.5 | 27.4 | 27.1 | 27.5 | 27.1 | 24.9 | 25.5 | 26.5 | 27.1 | 25.4 | 23.6 |
| Ntengo | | 17-Dec-13 | 22.9 | 23.9 | 23.5 | 23.7 | 23.5 | 21.9 | 24.0 | 23.0 | 21.7 | 23.2 | 22.5 | 21.7 | 23.5 | 23.2 | 21.6 |
| Ruace | | 20-Dec-13 | 25.2 | 24.7 | 24.5 | 23.9 | 24.3 | 24.8 | 24.8 | 23.1 | 25.2 | 24.4 | 23.2 | 24.1 | 24.9 | 24.5 | 23.3 |
| Sussundenga | | 21-Dec-13 | 25.8 | 24.9 | 24.2 | 25.5 | 24.2 | 25.0 | 24.2 | 26.5 | 25.7 | 24.7 | 24.3 | 25.0 | 25.0 | 24.1 | 21.2 |
| Brazil, crop season 2014/2015 | | | | | | | | | | | | | | | | | |
| Londrina | | 04-Nov-14 | 23.5 | 22.0 | 22.1 | 23.9 | 23.2 | 22.7 | 23.6 | 24.6 | 19.9 | 24.9 | 24.5 | 24.7 | 23.3 | 22.7 | 22.8 |
| Ponta Grossa | | 18-Nov-14 | 21.5 | 21.6 | 21.4 | 21.4 | 21.8 | 21.8 | 22.8 | 22.3 | 22.6 | 23.2 | 22.4 | 22.2 | 21.8 | 21.8 | 20.7 |
| Mozambique, crop season 2014/2015 | | | | | | | | | | | | | | | | | |
| Muriaze | | 05-Jan-15 | 25.8 | 25.8 | 25.5 | 26.2 | 25.8 | 25.1 | 26.1 | 26.0 | 25.6 | 26.0 | 22.9 | 25.2 | 26.7 | 25.7 | 25.5 |
| Ntengo | | 22-Dec-14 | 24.0 | 22.3 | 21.9 | 20.9 | 21.7 | 22.0 | 23.0 | 22.0 | 19.3 | 21.2 | 23.1 | 21.8 | 21.4 | 23.5 | 20.9 |
| Ruace | | 21-Jan-15 | 24.2 | 23.8 | 23.8 | 24.8 | 23.5 | 24.5 | 24.1 | 23.9 | 20.9 | 24.1 | 24.7 | 24.5 | 23.5 | 22.4 | 22.5 |
| Sussundenga | | 29-Dec-14 | 23.8 | 24.7 | 25.3 | 24.2 | 24.7 | 25.7 | 26.3 | 24.7 | 25.0 | 24.2 | 24.1 | 23.2 | 25.5 | 22.0 | 22.6 |

^1^As defined by Fehr and Caviness (1977). ^2^Due to logistic difficulties, temperature data were not recorded at Maracaí and Nkhame. Sowing dates were

23-Oct-13 at Maracaí; 20-Dec-13 and 23-Dec-14 at Nkhame.

**Supplementary Table 3**

Nodule number (NN, n° plant^-1^), nodule dry weight (NDW, mg plant^-1^), shoot dry weight (SDW, g plant^-1^), total N accumulation in shoots (TNS, mg plant^-1^), grain yield (GY, kg ha^-1^), grain dry weight (GDW, g 100 seeds^-1^), and relative effectiveness (RE, %) of soybean, cultivars BMX Potência – RR and BRS-359-RR, grown with or without inoculation treatment in the 2013/2014 and 2014/2015 crop seasons, across four sites (Londrina, Maracaí, Ponta Grossa and Rio Verde) in Brazil.

| Treatment^1^ | 2013/2014 crop season, across all sites | | | | | | |  | 2014/2015 crop season, across all sites | | | | | | | |
| --- | --- | --- | --- | --- | --- | --- | --- | --- | --- | --- | --- | --- | --- | --- | --- | --- |
|  | NN | NDW | SDW | TNS | GY | GDW | RE^2^ |  | NN | NDW | SDW | TNS | GY | GDW | RE^2^ | |
| NI | 18.2^a 3^ | 69.48^a^ | 1.5^c^ | 44.08^bc^ | 2126^c^ | 12.0^b^ | 84.7^b^ |  | 65.4^ns 3^ | 214.83^abc^ | 4.2^b^ | 167.88^cd^ | 2938^ns^ | 14.2^c^ | | 71.3^c^ |
| NI+N | 13.2^b^ | 38.50^b^ | 1.8^a^ | 58.74^a^ | 2538^a^ | 12.2^a^ | 100.0^4^ |  | 52.8 | 179.42^c^ | 6.0^a^ | 235.01^a^ | 3103 | 14.6^a^ | | 100.0^4^ |
| SEMIA 5079 | 19.0^a^ | 71.79^a^ | 1.5^cd^ | 45.84^b^ | 2178^bc^ | 12.2^a^ | 84.0^b^ |  | 66.5 | 244.24^ab^ | 4.5^b^ | 171.34^cd^ | 3110 | 14.2^bc^ | | 77.0^bc^ |
| SEMIA 5080 | 19.7^a^ | 67.05^a^ | 1.6^b^ | 46.83^b^ | 2070^c^ | 11.8^cd^ | 91.6^a^ |  | 61.1 | 199.79^bc^ | 4.2^b^ | 153.62^d^ | 3117 | 14.3^bc^ | | 71.0^c^ |
| SEMIA 587 | 17.4^a^ | 69.17^a^ | 1.4^cd^ | 44.63^bc^ | 2116^c^ | 12.0^bc^ | 82.0^b^ |  | 64.1 | 248.80^a^ | 4.5^b^ | 184.23^bcd^ | 3051 | 14.3^b^ | | 75.3^bc^ |
| SEMIA 5019 | 18.7^a^ | 63.75^a^ | 1.4^cd^ | 40.84^cd^ | 2117^c^ | 11.8^d^ | 77.9^bc^ |  | 73.8 | 232.98^ab^ | 4.9^b^ | 195.24^bc^ | 2991 | 14.1^c^ | | 84.4^b^ |
| USDA 110 | 16.8^a^ | 63.90^a^ | 1.3^de^ | 38.23^d^ | 2282^b^ | 11.9^bc^ | 73.1^c^ |  | 57.5 | 198.14^bc^ | 4.4^b^ | 177.11^cd^ | 3043 | 14.1^c^ | | 76.8^bc^ |
| 5079+5080 | 19.6^a^ | 69.77^a^ | 1.6^b^ | 47.39^b^ | 2203^bc^ | 12.1^a^ | 92.4^a^ |  | 62.7 | 216.16^abc^ | 5.7^a^ | 212.81^ab^ | 3059 | 14.4^b^ | | 95.5^a^ |
| *p -* value | 0.00 | 0.00 | 0.00 | 0.00 | 0.00 | 0.00 | 0.00 |  | 0.13 | 0.02 | 0.00 | 0.00 | 0.77 | 0.00 | | 0.01 |
| C.V. (%) | 16.93 | 17.28 | 7.70 | 8.09 | 5.83 | 0.93 | 7.81 |  | 18.98 | 17.30 | 14.75 | 16.09 | 6.58 | 1.36 | | 13.96 |

^1^ NI*,* No inoculation and no N-fertilizer applied; NI+N*,* no inoculation with 200 kg of N ha^−1^, split twice, applied at sowing and R2; SEMIA 5079*,* inoculated with *B. japonicum* strain SEMIA 5079; SEMIA 5080*,* inoculated with *B. diazoefficiens* strain SEMIA 5080; SEMIA 587*,* inoculated with *B. elkanii* strain SEMIA 587; SEMIA 5019*,* inoculated with *B. elkanii* strain SEMIA 5019; USDA 110*,* inoculated with *B. diazoefficiens* strain USDA 110; 5079+5080*,* inoculated with *B. japonicum* strain SEMIA 5079 and *B. diazoefficiens* strain SEMIA 5080; All rhizobia were applied at the rate of 1.2×10^6^ cells seed^−1^.

^2^ Determined as a ratio between the SDW of the inoculant and that of the treatment NI+N (Rufini et al., 2014).

^3^ Means of six replicates and when followed by same letter in the same column are not statistically different (*p ≤* 0.10, Duncan test).

^4^ Not included in the statistical analysis.

**Supplementary Table 4**

Nodule number (NN, n° plant-1), nodule dry weight (NDW, mg plant-1), shoot dry weight (SDW, g plant-1), above ground biomass (AGB, kg ha-1), grain yield (GY, kg ha-1), grain dry weight (GDW, g 100 seeds-1), and relative effectiveness (RE, %) of soybean, cultivar Storm, grown with or without inoculation treatment in the 2013/2014 and 2014/2015 crop seasons across five locations (Muriaze, Nkhame, Ntengo, Ruace and Sussundenga) in Mozambique.

| Treatment^1^ | 2013/2014 crop season, across all sites | | | | | | |  | 2014/2015 Crop season, across all sites | | | | | | |
| --- | --- | --- | --- | --- | --- | --- | --- | --- | --- | --- | --- | --- | --- | --- | --- |
|  | NN | NDW | SDW | AGB | GY | GDW | RE^2^ |  | NN | NDW | SDW | AGB | GY | GDW | RE^2^ |
| NI | 11.2^f^ ^3^ | 76.24^f^ | 20.7^c^ | 6689^bc^ | 2152^c^ | 14.8^e^ | 66.4^c^ |  | 13.8^e^ | 86.83^d^ | 15.5^e^ | 3506^d^ | 1110^c^ | 14.9^ns^ | 96.0^c^ |
| NI+N | 6.7^g^ | 43.47^g^ | 31.7^a^ | 6960^ab^ | 2581^a^ | 15.5^bc^ | 100.0^4^ |  | 10.6^f^ | 53.92^f^ | 19.3^abc^ | 3866^c^ | 1325^b^ | 15.8 | 100.0^4^ |
| SEMIA 5079 | 17.1^c^ | 180.43^c^ | 25.3^b^ | 6995^ab^ | 2481^a^ | 15.3^c^ | 80.1^ab^ |  | 25.4^c^ | 180.34^c^ | 20.7^a^ | 4073^bc^ | 1738^a^ | 15.0 | 118.3^ab^ |
| SEMIA 5080 | 25.2^b^ | 234.24^b^ | 23.7^bc^ | 7210^a^ | 2606^a^ | 15.6^b^ | 75.5^b^ |  | 28.7^b^ | 199.58^b^ | 18.2^bcd^ | 4320^ab^ | 1455^b^ | 15.3 | 122.6^a^ |
| SEMIA 587 | 14.8^d^ | 120.40^e^ | 25.5^b^ | 6576^c^ | 2265^bc^ | 15.3^c^ | 78.5^b^ |  | 26.1^c^ | 198.49^b^ | 19.7^ab^ | 4099^bc^ | 1382^b^ | 15.0 | 118.4^ab^ |
| SEMIA 5019 | 27.4^a^ | 295.45^a^ | 27.0^b^ | 7029^ab^ | 2527^a^ | 15.8^a^ | 87.8^a^ |  | 34.0^a^ | 255.85^a^ | 15.1^e^ | 4265^ab^ | 1374^b^ | 15.3 | 103.9^bc^ |
| USDA 110 | 12.8^e^ | 114.46^e^ | 24.5^b^ | 6835^abc^ | 2342^b^ | 15.0^d^ | 75.7^b^ |  | 18.2^d^ | 68.98^e^ | 16.1^de^ | 4200^ab^ | 1633^a^ | 15.3 | 99.8^c^ |
| *p -* value | 0.00 | 0.00 | 0.00 | 0.00 | 0.00 | 0.00 | 0.01 |  | 0.00 | 0.00 | 0.00 | 0.00 | 0.00 | 0.14 | 0.04 |
| C.V. (%) | 7.98 | 10.31 | 12.4 | 4.78 | 5.35 | 1.22 | 9.81 |  | 9.33 | 6.91 | 11.50 | 6.19 | 8.22 | 3.14 | 12.99 |

^1^ NI*,* No inoculation and no N-fertilizer applied; NI+N*,* no inoculation with 200 kg of N ha^−1^, split twice, applied at sowing and R2; SEMIA 5079*,* inoculated with *B. japonicum* strain SEMIA 5079; SEMIA 5080*,* inoculated with *B. diazoefficiens* strain SEMIA 5080; SEMIA 587*,* inoculated with *B. elkanii* strain SEMIA 587; SEMIA 5019*,* inoculated with *B. elkanii* strain SEMIA 5019; USDA 110*,* inoculated with *B. diazoefficiens* strain USDA 110; All rhizobia were applied at the rate of 1.2×10^6^ cells seed^−1^.

^2^ Determined as a ratio between the SDW of the inoculant and that of the treatment NI+N (Rufini et al., 2014).

^3^ Means of five replicates and when followed by same letter in the same column are not statistically different (*p ≤* 0.10, Duncan test).

^4^ Not included in the statistical analysis.
